# 

**Published:** 1846-03

**Authors:** 


CONTENTS:

Cure of Encysted Dropsy by exciting irritation in the Sac. - 178

MEDICAL INTELLIGENCE.

Epidemic Erysipelas. By G. N. FITCH, M. D. - - - 179

Medical Convention. - - 180

PRACTICAL MEDICINE, &c..

The Discussion on the treatment of Placenta Prævia. - - 182

Clinical Researches on the exhibition of Kermes Mineral in Pulmonary affections. - - 186

